# Resonance excitation of surface capillary waves to enhance material removal for laser material processing

**DOI:** 10.1038/s41598-019-44577-6

**Published:** 2019-05-31

**Authors:** Sonny Ly, Gabe Guss, Alexander M. Rubenchik, Wesley J. Keller, Nan Shen, Raluca A. Negres, Jeff Bude

**Affiliations:** NIF and Photon Sciences, Lawrence Livermore National Laboratory, 7000 East Avenue, Livermore, CA 94550 USA

**Keywords:** Materials science, Techniques and instrumentation

## Abstract

The results of detailed experiments and high fidelity modeling of melt pool dynamics, droplet ejections and hole drilling produced by periodic modulation of laser intensity are presented. Ultra-high speed imaging revealed that melt pool oscillations can drive large removal of material when excited at the natural oscillation frequency. The physics of capillary surface wave excitation is discussed and simulation is provided to elucidate the experimental results. The removal rates and drill through times as a function of driving frequency is investigated. The resonant removal mechanism is driven by both recoil momentum and thermocapillary force but the key observation is the latter effect does not require evaporation of material, which can significantly enhance the efficiency for laser drilling process. We compared the drilling of holes through a 2 mm-thick Al plate at modulation frequencies up to 20 kHz. At the optimal frequency of 8 kHz, near the resonant response of the melt pool, the drilling efficiency is greater than 10x with aspect ratio of 12:1, and without the collateral damage that is observed in unmodulated CW drilling.

## Introduction

Laser metal drilling and cutting involves melting the metal and removing the molten liquid from the hole. Typically the removal is performed by a pressurized gas stream but this removal mechanism is difficult to use when the drill hole is small (~mm). In this case, the melt is removed by the recoil pressure produced by the metal vapors ejected from the heated surface. During melting, the temperature of the metal surface approaches and exceeds the boiling temperature T_b_, and a metallic vapor jet is formed. Recoil pressure generated by the vapor expansion produces a downward force on the melt pool causing rapid melt pool motion leading to liquid melt being ejected away^1–3^. For effective material removal, a large area of the metal surface must be heated to above T_b_, requiring significantly more energy than required to melt the surface. For example, aluminum has a T_b_ ~ 2,730 K nearly three times higher than the melting point T_m_ ~ 933 K which requires raising the laser power by the same factor (varies linearly with T_b_/T_m_). At high laser power, other highly undesirable effects occur. The intense vapor flux absorbs the laser beam and shields the surface, further reducing the efficiency. In addition, the undesirable overheating affects the cooling process and can lead to cracking on the metal surface and inside the hole walls, degrading the process quality.

On the other hand, the melt pool supports capillary surface waves which oscillate with a wide range of different frequencies related to the melt pool and keyhole geometry^4–6^. The oscillations can be excited by an external driver such as mechanical and acoustic waves^7^, electrical current^8^, or laser irradiation^9^ and are akin to the well known ripple effect of a droplet impinging on a liquid surface^10,11^. Since the melt has a low viscosity, the weakly damped capillary surface waves can be easily excited by recoil pressure and thermocapillary forces. Growth of the waves can induce large melt motion and result in melt ejection at temperatures below T_b_. Surface wave oscillations has been investigated extensively in arc welding for a wide range of conditions^12–19^ while studies in laser processing are more limited^5,20^. Typically, the laser-melt pool sizes are small (10’s to 100’s µm) with oscillation frequencies in the kHz regime. Detecting these high frequency oscillations requires advanced diagnostics with µm spatial resolution and µs temporal resolution which makes exploring this phenomenon difficult.

In this paper we propose an efficient removal mechanism based on resonant excitation of surface capillary waves using periodic modulation of a laser intensity. We demonstrate that the optimal selection of modulation frequency can greatly enhance the melt removal volume and enables removal at lower temperatures, even in the absence of strong recoil pressure. If the modulation frequency matches the natural oscillation of the liquid melt, a resonance effect will be produced, and the oscillation amplitude will peak, thus leading to large droplet ejections; in contrast when the modulation is detuned from the natural frequency, the removal efficiency drops sharply.

One of the earliest experiments with modulated laser intensity was performed using chopped radiation of a pulsed laser to irradiate 180 µm steel blade^21^. The authors noticed enhanced removal of material in the kHz regime and suggested that oscillation modes can play a role in ejection but did not provide any detailed explanations. Melt pool surface waves effects were studied in welding for common metals and have been shown to reduce metal porosity^22^, prevent hot cracking^23^ and increase keyhole stabilities^24^. While evidence was presented in some of these studies to support the positive effects of periodic modulation, the underlying physics and broader impact on other observables, most notably molten liquid ejection, is far from complete. Although the physics involved is somewhat specific to drilling technology and laser additive manufacturing processes, a better understanding of capillary wave excitation and melt oscillations can help shed light in other applications.

The experiments presented here were conducted with Aluminum alloy 6061 (Al6061). We will describe the resonance frequency dependence of the removal rate, the coupling mechanisms of the surface waves with the processing laser beam, and the material ejection process. Experimental evidence is provided via high speed imaging at up 200 kfps. To help elucidate the experimental results, the laser-melt pool interaction is modeled with a multi-physics finite element code (ALE3D)^25^. The combination of the experimental and computational results provides a holistic picture of the hydrodynamic effects related to laser metal drilling. Finally, as an application, we demonstrate the drilling of small <300 µm holes in 2 mm-thick Al plate. We show that a 10x higher drilling efficiency can be obtained and without the collateral damage observed in unmodulated CW drilling.

## Results and Discussion

### Resonant oscillation of the melt pool

Let us estimate the natural oscillation frequency of the capillary wave. In an ideal fluid, the angular frequency of the capillary wave on a free surface is given by the dispersion relation^26^:

1 $${{\omega }_{0}}^{2}=\frac{\sigma {\kappa }^{3}}{\rho }$$

where σ is the surface tension, k is the wave number of the capillary wave, and ρ is the liquid metal density. For a simple estimate, we can assume that the melt pool has a planar geometry with melt diameter *a*, and the lowest order mode can be approximated as k~1/*a* (this is the lowest order axi-symmetric vibration). The frequency increase with wave number and decreases with melt diameter. For Al experiments with a = 50 µm, *σ* = 0.9 N/m and *ρ*=2.35 g/cm^3^, the frequency is then ν = ω_0_/2π ~9 kHz. In a viscous fluid, *ω* = *ω*_0_ + i*γ* where the damping coefficient *γ* ≈ 2*μk*^2^. For viscosity *ω* ~10^−2^ cm^2^/s (from ref.^26^),*γ* ≈ 800 *Hz* which is small in comparison with the resonant frequency at 9 kHz (*γ*/***ω***_o_<< 1) – that is, the melt pool is a weakly damped oscillator.

The calculation above is based on planar geometry for shallow ablation pits and is not representative for conditions in deep drilling where constant changes in melt pool geometry continuously alters the frequency of surface waves. More sophisticated analytic models have been developed to calculate the eigenmodes of oscillations based on the complex geometry of the melt pool^5,6,20,27,28^.

### Effect of modulation frequencies on removal rate for ablation pits

**Figure 1:**
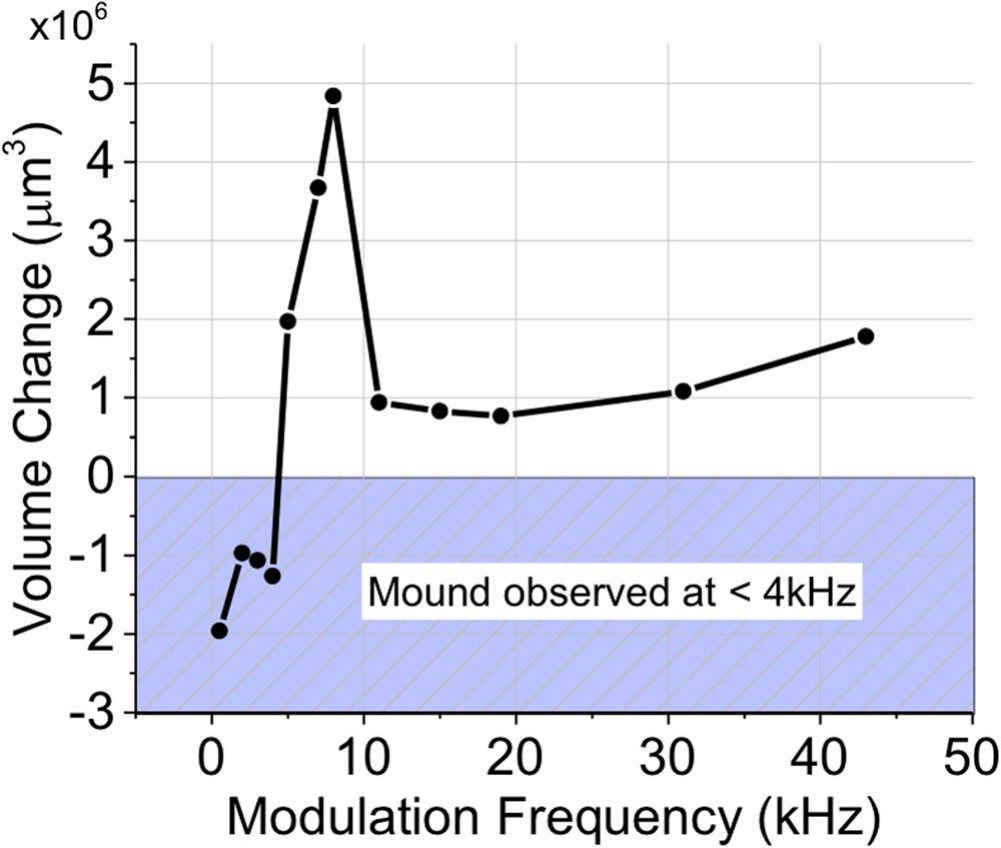
Volume removed vs frequency at 5 ms drill time. The largest volume was removed at 8 kHz. Negative values on y-axis indicate a mound at the surface due to material pileup. Beam diameter was 75 μm 1/e^2^. At 0 kHz, the average power is 300 W, and with modulation the average power is 300 W with peak power 600 W.

An array of laser exposures with a total exposure time of 5 ms and varying frequencies of modulation were produced on 2 mm thick Al plate. At the melt peak temperature T_p_~2–3*T_m_, the melt diameter is about the scale of the 1/e beam intensity modulation. For a 75 µm 1/e^2^ beam used here, this is approximately 50 µm 1/e melt diameter (divide by $\sqrt{2}$). The average power was 300 W with 600 W peak power. The volume of the pits was measured with a confocal microscope (Keyence VK-X100) and plotted as a function of frequency in Fig. 1. From Fig. 1, the removal can be separated into three distinct regions. For modulation frequency *ω*_M_ <4 kHz, no resonance effect occurs, and in fact, the pit volume is negative due to a mound formation at the surface from thermal expansion and pileup of material when solidified. For an unmodulated pump at P = 300 W average power, the material removal is absent since the recoil momentum is too weak to overcome the surface tension and eject the liquid droplets. For *ω*_M_ between 5 and 8 kHz, ablation pits were formed, and the volume removed rapidly increases to a peak at 8 kHz before decreasing from 8 to 10 kHz. The peak removal at 8 kHz is near the resonant response of the material as predicted in equation (1). For *ω*_M_ >10 kHz, the removal is approximately constant and indicates that the ablated volume becomes independent of the modulation. This is likely due to the broad resonance at high frequencies which supports excitation of higher order modes.

### Coupling of laser intensity modulation to surface capillary waves

Here we will describe the specific mechanisms of the coupling of laser intensity modulations to the amplification of the capillary surface waves. First, these waves can be excited by both laser recoil pressure and thermocapillary forces and depends on the temperature of the melt spot. The steady state temperature distribution produced by 1/e^2^ Gaussian beam diameter *d* is^1^:

2 $$T=\frac{AP(t)}{dk\sqrt{\pi }}$$

For Al, the absorptivity of common industrial samples is A = 0.2 (from ref.^29^), and the thermal conductivity is *κ* = 0.02 W/m*K. For a beam diameter d = 50 µm at P = 300 W, the peak temperature of the melt generated at center of the beam is ~3,000 K, slightly above the boiling temperature ~ 2,730 K, and weak recoil pressure is generated. Away from the beam center, the temperature of the melt can drop below boiling. The recoil pressure Pr at a surface heated to temperature T is given by expression:

3 $$Pr=0.5P{r}^{\ast }\exp [\lambda (\frac{1}{{T}_{b}}-\frac{1}{T})]$$

here Pr* = 10^5^ Pascal is the atmospheric pressure, λ is evaporation enthalpy per atom (3 eV for Al) and T_b_ is the boiling temperature. For T = 3000 K, P = 1.6 Pr*. To eject melt, the recoil pressure must overcome surface tension Pr > 2σ/R, where R is the local melt pool curvature radius and σ the surface tension (for Al σ~0.9 N/m). If we assume R as approximately the laser beam radius d/2, Pr must exceed 0.7*Pr* to drive liquid flow. Physically though, R is smaller because the recoil pressure is localized near the beam center, and the recoil pressure required to overcome the surface tension is effectively larger. For a small area around center of melt, the pressure produced by the surface tension dominates and little melt ejection is expected. At temperature well above boiling temperature, then recoil pressure become strong due to the exponential temperature dependence.

However, there is another mechanism of exciting capillary surface wave that can operate even at temperature below the boiling point based on the thermocapillary effect^30^. The surface tension of metals σ(*T*) is usually temperature dependent. During melting, the laser creates a high temperature gradient $\frac{\partial T}{\partial r}$ across the melt spot, which in turn imparts a thermocapillary stress that drives the melt in motion tangentially. The thermocapillary stress is given by:

4 $$\frac{\partial \sigma }{\partial r}=\frac{\partial \sigma \partial T}{\partial T\,\partial r}$$

In general, for metals $\frac{\partial \sigma }{\partial r} < 0$ and the stress drives the melt out from the center of melt spot. For our parameters the modulation period ~125 µs is long in comparison with thermal diffusion time $t=\frac{{d}^{2}}{4D} \sim 5\,\mu s$ (where D =1.03 cm^2^/s is the thermal diffusivity of Al). The thermal distribution is considered quasi-stationary with the maximum temperature of the melt induced at the center of beam r=0. At this point, $\frac{\partial T}{\,\partial r}$ is zero. This interestingly implies that the thermocapillary stress is zero at beam center (and also zero towards the tail end of the beam). The maximum stress depends on T(r), which is a complex function that depends on the melt pool geometry^1^, but qualitatively the maximum is near r~d/2. The distribution of the driving stress matches better with the spatial distribution of the higher order modes, that is there is a good probability of exciting other eigenmode oscillations.

For a modulated beam, the power fluctuates sinusoidally:

5 $$P(t)={P}_{avg}+{P}_{amp}\,\sin ({\omega }_{M}t)$$

where P_avg_ = 300 W is the average power, P_amp_ = 300 W is the amplitude power and ω_M_ is the frequency of modulation. The temperature changes with the sinusoidal variation in power T~P(t). During the ramp up of the power cycle, the surface temperature is maximum at peak power of 600 W. Both recoil momentum and thermocapillary stress applied to the surface can excite capillary waves and lead to material ejection. During the ramp down to a minimum of 20 W (minimum lasr operating power), the temperature drops below T_b_ and the recoil pressure ceases. We can imagine the sinusoidal modulation as a periodical piston applied at the melt center. As we will show later from simulations and hole drilling experiments, thermocapillary stress from the periodic modulation, can drive significant material removal. If the period of the modulation matches with the resonance of the capillary surface waves, the material removal rate is enhanced. The mechanism of surface wave excitation can work even when the peak temperature in the melt pool is below the boiling point.

### High speed Imaging to visualize material ejection

**Figure 2:**
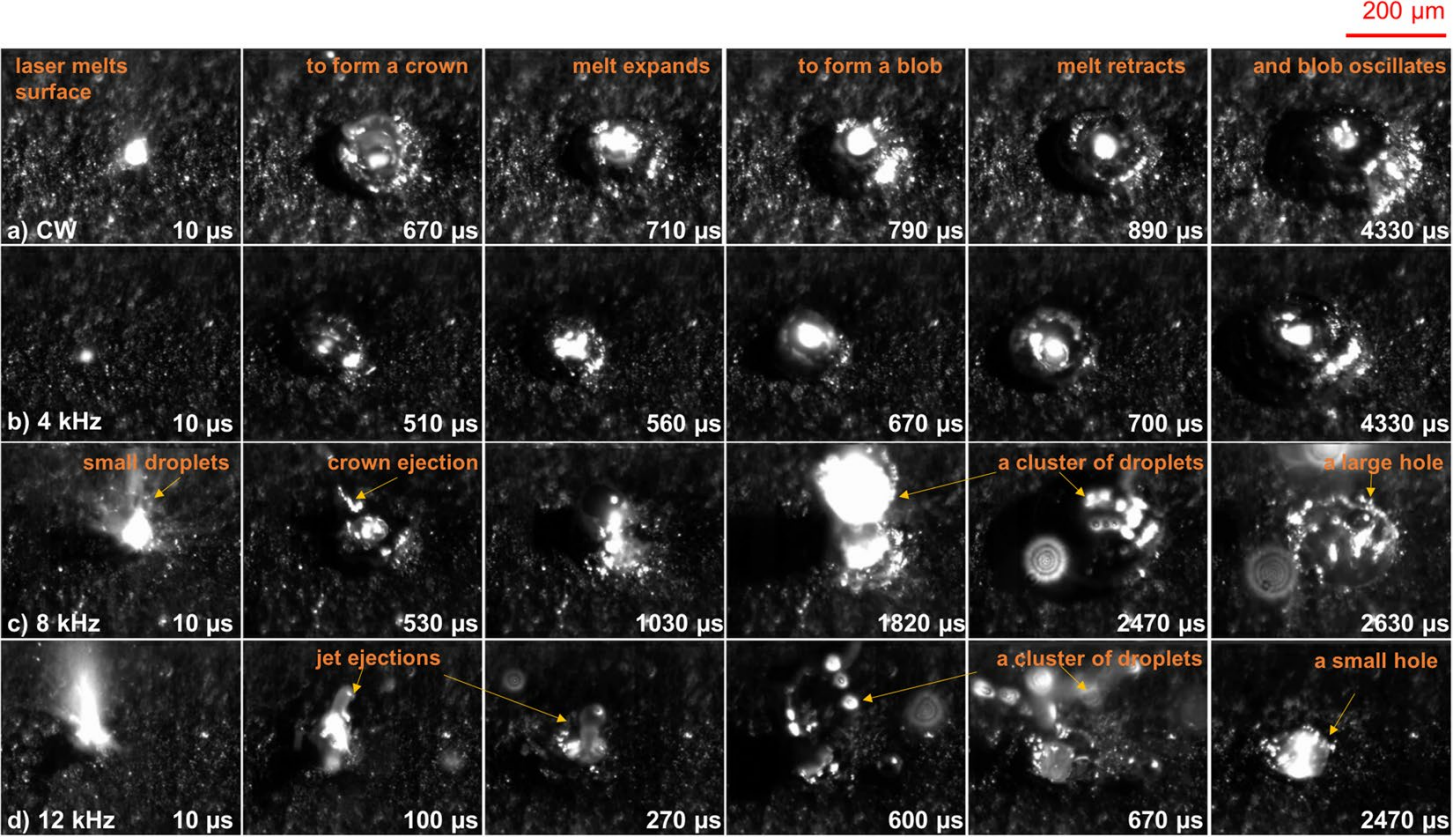
Comparison of different modulation frequencies during laser drilling recorded at 100 kfps. (**a**) Unmodulated CW (0 kHz) - shows a typical melt oscillation cycle and low amount of ejections. (**b**) 4 kHz – similar sequence of events as in (**a**). (**c**) At 8 kHz, many different types of ejections are observed. Small droplets at 10 μs, crown ejection at 530 μs, and a large cluster of droplets at 1820 μs and 2470 μs. A large ablation hole is formed at 2630 μs. (**d**) At 12 kHz, similar ejection mechanism as case (**c**) with jet ejections at 100 μs and 270 μs, large cluster of droplets at 670 μs, and formation of a smaller ablation hole at 2470 μs. The drill time for each experiment is 5 ms. The average power is 300 W with peak power at 600 W. Full length video can be found in Supplementary Movie 1.

To understand the mechanism of removal under modulated laser exposure, we used high speed imaging at 100 kfps to compare the oscillations at CW and 4 kHz with the oscillations at 8 kHz and 12 kHz, near the resonant response of the material. The average power is 300 W for the CW case, and the peak power is 600 W for the modulated case. The total drill time is 5 ms. Figure 2 illustrates six snapshots taken from the start of the laser (full video is provided as Supplementary Movie 1). In all four cases, the fluctuations are chaotic for the first few oscillation cycles (up to 700 μs on the video frames) with a small amount of visible surface modulation. Recoil momemtum pushes down on the melt and force the melt to flow towards the side. At the same time, thermocapillary forces act along the surface and also drives the melt from from the center to the side. Both mechanism results in the melt to form a crown like shape. During melting, the material density decreases (from 2.7 to 2.35 g/cm^3^ for Al), and now the same amount of material occupies a larger volume. The volume expands upward but surface tension pulls the melt inward to form a blob. For the case of CW (and similarly at 4 kHz), the liquid blob stops expanding laterally since the melt does not wet the substrate, and subsequently retracts. As a result, the blob is stable – the nonresonant excitation of the capillary waves induced only oscillations with little melt ejection. The cycle repeats itself every 100–150 μs with a blob that moves up and down (axi-symmetric mode). Furthermore, at 0 kHz, the resulting temperature at 300 W is too low to produce recoil momentum sufficient for large melt removal. Thermal expansion of the material leads to pileup consistent with Fig. 1.

For modulation frequencies at 8 kHz and 12 kHz, near the resonance, large melt removal is observed throughout the entire drilling time. Small, high velocity droplets eject within 5 μs of the laser initiation. As the crown expands, droplets also break apart from the perimeter of the crown. The depression formed is now large enough that during melt retraction, a central jet emerges from the center of the pit (at 100 μs and 270 μs in Fig. 2d) and escapes in the vertical direction. Unlike the CW case where liquid is constantly ejected, in the near resonance case, periodic burst of materials occurs every few cycles due to the time it takes for the resonance to build up. At 2470 μs in Fig. 2c and 670 μs in Fig. 2d, a large cluster of particles explode violently outward to leave a hole. As the laser penetrates deeper into the channel, a large jet of liquid bursts out (see video at 3700 μs and 5300 μs at 8 kHz). From the video, both axi-symmetric (up and down) and asymmetric (sloshing mode) oscillations are observed.

Supplementary Movie 2 provides a very clear visualization of the resonance effect at 8 kHz. The video starts recording at 1 ms after the laser turns on, when the melt pool is already formed. The first fame is reset to time 0 μs. During cycle 1 (125 μs), the melt blob oscillates up and down, and during cycle 2 (250 μs), the amplitude of the oscillation has increased. By the third cycle, the amplitude peaks out and a ring shaped melt ejects away (400 μs) leaving a hole. From analyzing multiple videos, it takes anywhere from 3 to 6 cycles for the waves to transfer energy to the thermocapillary stress and eject material. This leads to large number of bright ejections from time to time. The amplification of the oscillations requires good symmetry of the melt flow but this symmetry can be broken by excitation of higher order modes (such as an asymmetric sloshing mode) which disrupts the growth of the surface waves.

Supplementary Movie 3 provides a sideview of the ejection process at 8 different frequencies: 300 and 600 W CW, 2 kHz, 4 kHz, 8 kHz, 12 kHz, 16 kHz, and 20 kHz. This movie uses a large field of view (~cm’s) to visualize the number of particles emitted, the ejection velocity, and brightness relative to other modulation frequencies. The intensity scale of the eight frequency panels are kept the same for direct comparison. Video is recorded at 50 kfps. At frequencies below 4 kHz, the ejection rate and intensities are low and in particular, for the 2 kHz case, the ejections are barely visible. Above 8 kHz, many bright droplets are ejected with burst of large number of particles followed by several ms of arrest (see for instance between 7.5 to 8.5 ms at 8 kHz). At 600 W CW, the temperature of the melt surface is more than two times T_b_ and nearly twice that of 300 W, and this results in constant small ejections. Also notable in the video is particle entrainment in the presence of Argon gas, similar to the particle motion observed in selective laser melting^3^.

### Multi-physics hydrocode simulation of modulated power CW laser drilling

In order to investigate the relevant physical processes that drive enhanced material removal efficiency during modulated power CW laser drilling, we performed 3D laser-material interaction (LMI) simulations using LLNL’s multi-physics hydrocode ALE3D^25^. ALE3D is an arbitrary Lagrangian-Eulerian finite element code with coupled hydrodynamics and thermal diffusion solvers (explicit and implicit) for modeling heat transfer, phase transformation, and the fluid and elastic-plastic response of materials on an unstructured grid. For LMI simulation, laser energy deposition is modeled with a ray casting laser package that accounts for evolving surface topology and nonlinear absorptivity. Material models for the study were developed using experimentally anchored multi-phase equation of state (EOS) models derived from the Livermore Equation of State (LEOS) database, along with experimentally calibrated Steinberg-Guinan models for constitutive response. Nonlinear temperature-dependent models for ablative recoil pressure and surface tension, tracked and imposed along the material interface, were modeled based on^31^ and^32,33^ respectively.

**Figure 3:**
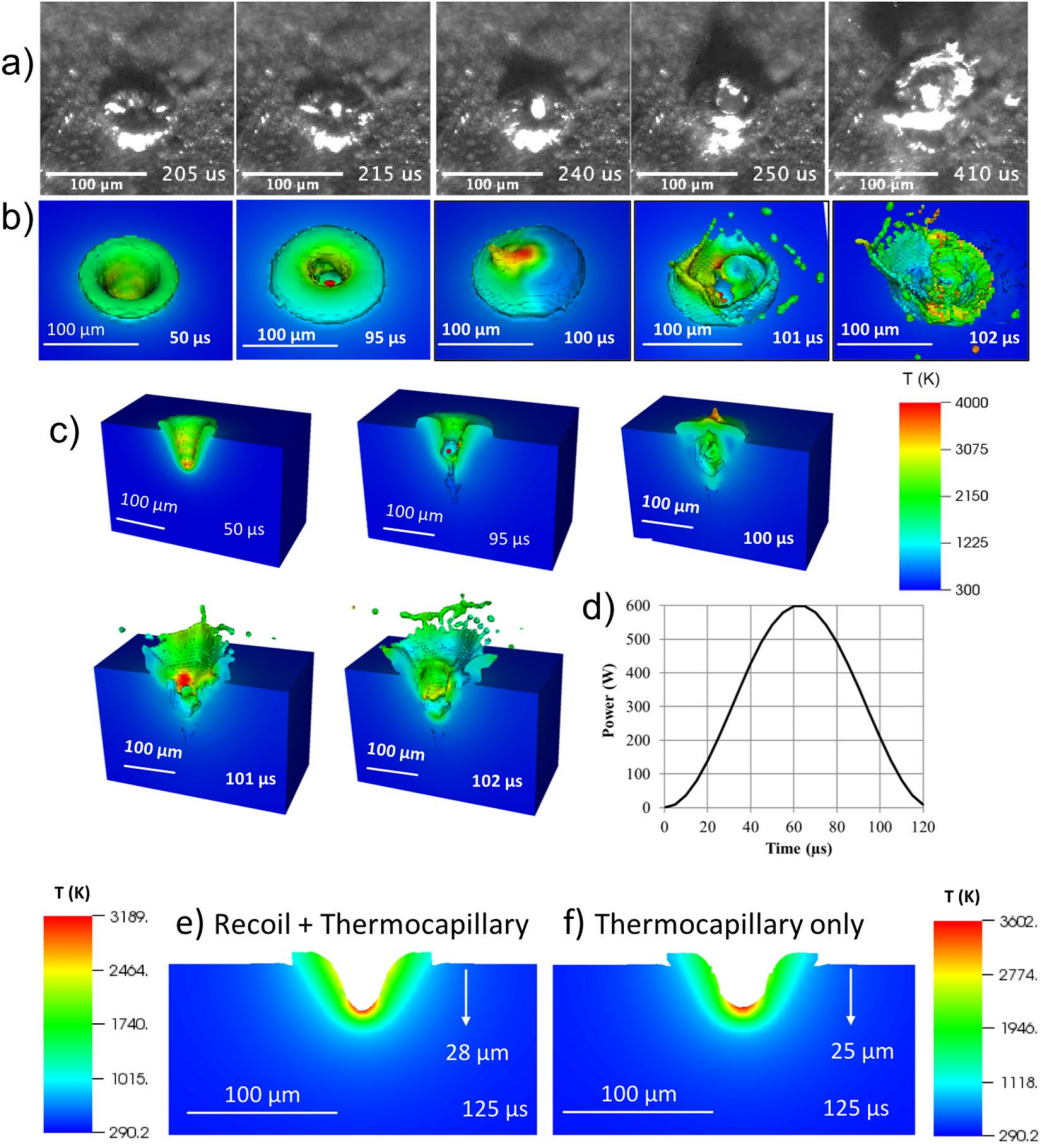
Comparison of ALE3D modulated CW laser drilling simulation with experiment (Al 6061, 8 kHz, 300 W average power, 600 W peak power, 50 μm beam diameter). (**a**) Five frames from high speed video showing melt oscillation at 8 kHz over approximately 2 cycles. Videos are recorded at 200 kfps and provided in Supplementary Movie 2. ALE3D simulation showing depression formation (50 μs) during power ramp up, melt inflow (95 μs) and depression collapse (100 μs) during power ramp down, and material ejection (101–102 μs with **a**,**b**) surface view and (**c**) mid-plane view. (**d**) Power modulation cycle. (**e**,**f**) Instantaneous melt depression depth for recoil pressure and thermocapillary force and for thermocapillary force only, respectively (Al 6061, 12 kHz, 300 W average power, 600 W peak power, 80 μm beam diameter).

Figure 3 provides a comparison of the melt dynamics predicted by hydrocode simulation with high speed video from the experiment for an Al 6061 specimen irradiated with an 8 kHz modulation, 300 W average power and 600 W peak power CW laser focused to a 50 μm 1/e^2^ diameter spot. The simulations utilized a ray casting laser energy deposition model, which tracks surface evolution, and assumed a constant absorptivity of 20%.

The first four images in Fig. 3a shows the melt pool oscillation through one cycle of power modulation (same as Supplementary Movie 2). The last image shows a ring-like ejection at the next cycle (410 μs). The images depict both the oscillatory response of the melt during the power modulation cycle, as well as material out-jetting and splashing near the minima of the power history. During the ramp in laser power, recoil pressure and thermocapillary force (nonuniform temperature-dependent surface tension) lead to the formation of a melt depression (50 μs), which reaches a peak depth around 70 μs (image not shown). As laser power ramps down and the surface cools through evaporative, convective, and radiative heat loss, the melt flows back into the depression. Depending on the depth of the depression and the power reduction rate (which influences surface temperature and, therefore, ablative recoil pressure and thermocapillary force), this inflow of material during collapse of the depression can drive out-jetting of material and splashing, as observed in both the experiment and simulation. Figure 3b,c shows surface and mid-plane section views from the hydrocode simulation, overlaid with material temperature. The hydrocode simulation qualitatively reproduces the processes of depression formation (50 μs) during the ramp in power, melt inflow (95 μs) and depression collapse (100 μs) during the ramp down in power, and the ensuing material ejection (101–102 μs), including out-jetting and splashing. Figure 3d shows the power modulation cycle used in the simulations. The melt temperature caps out at 4,000 K but most of the melt flow and removal happens below 3,000 K.

Removal of material through this hydrodynamic melt ejection mechanism (out-jetting and splashing) is efficient from an energy standpoint in that it requires significantly less energy than equivalent removal through vaporization. The recoil pressure for Al remains relatively low near the boiling point, where advective transport within the plume maintains low pressure at the ablation surface^31^. In contrast, surface tension capacity drops steadily above the melting point^33^. As the near surface temperature is driven above ~4000 K, well above the approximate boiling point of 2700 K, there is an exponential increase in the recoil pressure due to the build up of higher temperature ablation products near the surface. Thus, the relative impact of ablative recoil pressure and thermocapillary effect depends on the heating condition, with the former dominating when near surface temperatures are driven well above the boiling point and the latter driving melt flow at lower temperature.

The simulations demonstrate that nonuniform temperature-dependent thermocapillary effects is sufficient to drive the melt within the hot laser spot to the colder outer regions to form a depression in the absence of strong recoil pressure. This is illustrated in Fig. 3(e,f) for the case of an Al 6061 specimen irradiated with a 12 kHz modulated, 300 W average power laser focused to an 80 μm 1/e^2^ diameter spot. For this case, an absorptivity of 10% was utilized. The instantaneous melt depression depth at 125 μs (near peak power during the second power modulation cycle) is shown in Fig. 3e for the combined effect of ablative recoil pressure and thermocapillary force (28 μm), and in Fig. 3f for thermocapillary force only (25 μm). There is ~10% increase in melt depth with addition of recoil pressure. At this point in time, the models do not have sufficient fidelity to capture near-resonant amplification which can drive the process toward instability. The simulation supports the experimental result that the mechanism of surface wave excitation can work even when the peak temperature in the melt pool is below the boiling point.

### Applications to laser hole drilling

**Figure 4:**
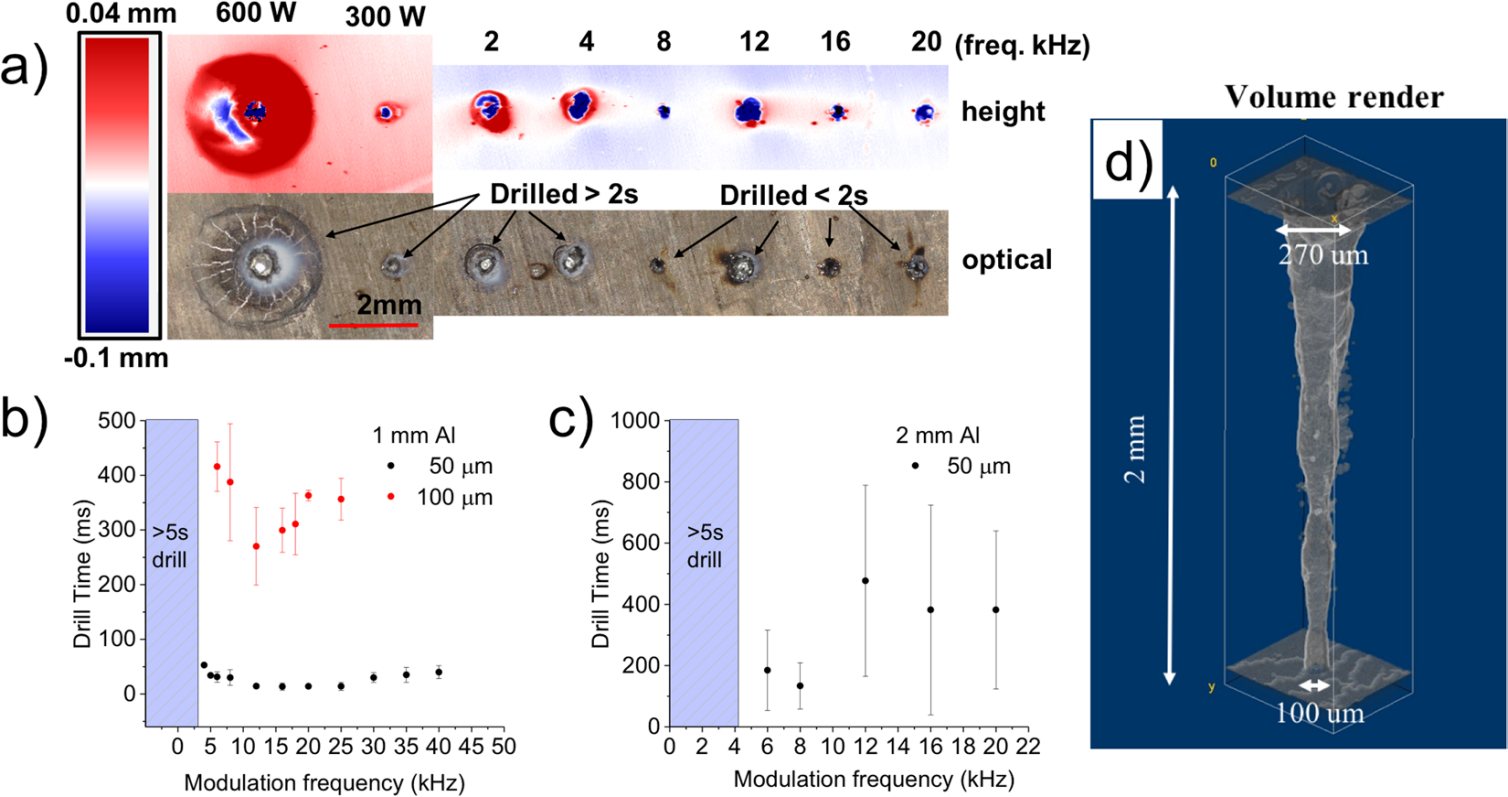
(**a**) Entrance hole images for 2 mm thick aluminum at different modulation frequencies with a 50 μm beam. Top panel is the height/depth image with modulation frequencies listed. The 600 W and 300 W sites were created with the unmodulated CW beam. The bottom panel is an optical image with drill times listed. Drill time for 1 mm Al is shown in (**b**) with optimal removal at ~12 kHz. The red symbols correspond to the 100 μm beam, and the black symbols to the 50 μm beam. (**c**) Drill time for 2 mm thick Al corresponding to the images on (**a**) with optimal removal is at ~8 kHz. (**d**) CT scan of a typical channel created at 8 kHz. For (**b**,**c**), the average power with modulation is 300 W with a peak power of 600 W, and the average power is 300 W for unmodulated case.

Previously we discussed the material removal for shallow ablation pits after a 5 ms laser irradiation. Here a modulated beam was used to drill holes in a 1- and 2-mm-thick aluminum plate to demonstrate a practical application of this technique. We chose aluminum alloy Al6061 since this material is difficult for laser processing due to its high thermal conductivity and reflectivity We show that drilling of holes is possible even when the melt temperature is below T_b_. Figure 4a shows the confocal image and height profile of the entrance hole at various frequencies for the 2 mm sample. For the unmodulated CW at 600 W, the entrance hole is large ~700 µm surrounded by a large heat affected zone with multiple cracks. The collateral damage (i.e. cracking, material redeposition, large heat zone) produced by CW excitation is well known. In some cases we had to stop the experiment since the 1″ diameter plate began to fully melt and sag out of the target holder, which is common for high thermal conductivity material like Al. For the modulated beam, the entrance holes are smaller, and the heat affected zone is practically absent. Videos of the different frequency panels in Supplementary Movie 3 corresponds to the confocal images shown in Fig. 4a.

For a 1 mm sample using a d=50 µm 1/e^2^ modulated beam with peak power at 600 W, the drill- through time is around 20 ms, reproducible from test to test, with maximum removal around 12 kHz (Fig. 4b). When the beam size is doubled to d=100 µm 1/e^2^ at the same power P, the drill- through time increased above 200 ms. From Equation (2), doubling the beam size at the same power lowers the melt temperature at surface by half from 6,000 K to 3,000 K (or equivalently, the temperature produced by a 300 W CW beam). Yet with the larger beam size, we were still able to drill holes, with an optimal removal also centered around 12 kHz, which shows that laser drilling is possible below T_b_ where recoil momentum is not sufficient to overcome the surface tension and drive melt flow. Based on Equation (1), there should have been a shift towards lower resonant frequency, but that equation assumes a planar geometry and is not representative for conditions in deep drilling where constant changes in melt pool geometry continuously alters the frequency of surface waves. In addition, during the modulation period, the intensity swings from high to low, which also changes the melt pool size, and can shift or broaden the resonant frequency. There is also some variation in drill-through time likely related to the decrease of melt temperature (T~P/d). At frequencies below 4 kHz, we were not able to drill through after 5 s and stopped the experiment.

For the 2 mm sample, the 50 µm modulated beam always drilled through in less than 1 s, but drill-through times varied stochastically with a trend to optimum removal at 8 kHz (Fig. 4c). At frequencies below 4 kHz, again we did not observe drill through after 5 s. There is a large dispersion in drill-through times. This is not surprising since there are key differences between ablation of shallow pits and drilling of deep holes^34^. First, as the laser drills deeper into the channel, many effects come into play. The laser radiation is reflected, diffracted and absorbed by the walls. Multiple reflections inside a keyhole or in deep drilling processes can produce the hot spots down the axis of the channel, and consequently the local intensity can be very high, leading to local boiling and material ejection^35^. However intensity modulation should be independent of the multiple reflections effect yet we see a large a large difference in removal rate between modulated and unmodulated pump. Hence, we believe that thermocapillary effects plays a more important role in material removal for the processing parameters used here. Second, the melted layer has to travel along the walls back up the hole entrance but surface tension can stop it. The melt retracts back towards the center of the channel and pushes upward to form a central jet that tries to escape in the vertical direction (as observed in Fig. 2 for the 12 kHz case at 100 µs and 270 µs). This attempt to escape often fails which slows down the material removal rate. And third, Fourier analysis and time resolved absorptance measurements have shown that keyhole oscillations can also shift or broaden the resonant frequency at the melt surface^4,36^. The combination of these effects contribute to the dispersion in drill-through times.

A computed tomography CT (Zeiss Xradia 510 Versa) rendering of a typical hole drilled at 8 kHz is shown in Fig. 4d. The hole has an entrance size of 270 μm and an exit diameter of 100 μm. The entrance cone is 70 degrees and the overall taper is 87.5 degrees. The volume, V, of the hole is 45×10^−6^ μm^3^. The aspect ratio is 12:1 (based on the average diameter in the hole) but aspect ratios of 20:1 are achievable depending on modulation frequency. An important, additional distinction compared to unmodulated CW drilling is that the heat affected zone is practically absent. Little surface debris and resolidified material is present, and the hole would be of high quality as judged by the metrics defined in ref.^37^.

The absorptivity of pure Al is about 5% for 𝜆=1 µm at 300 K (from ref.^38^) whereas solid industrial grade Al can be four times higher due to increase in surface roughness and the presence of an oxide layer^39^. However, in laser drilling, surface tension helps by smoothing out the wall roughness and decreasing the absorptivity. Vapor ejected from the hole subsequently interacts with the air to prevent it from entering the hole and oxidizing the walls. Thus, based on the quality of the hole revealed by the CT scan, it is likely that oxidation effects are mitigated and thus the light is channeled through the hole with low losses.

The amount of energy used to drill through a 2 mm sample with modulated beam is E=60 J (E =  Power * drill-through time  =  300 W * 0.2 s). In contrast for the unmodulated beam, this requires at least 3 kJ (600 W* 5 s). The volumetric removal efficiency using modulation is E/V ≈ 1.3 MJ/cm3 which is 12x more efficient than the unmodulated CW beam (assuming the hole diameter is 2x larger for CW). We emphasize that modulated drilling is not just more efficient, but the quality of the hole is better with smaller heat affected zone and less collateral damage. Given the recent advances in high powered fiber laser, this technique offers a more cost effective alternative than short pulse laser drilling.

## Conclusion

In this study, we have presented experimental and simulation results to demonstrate that resonant excitation of surface capillary waves can enhance material removal rate by more than 10x. The mechanism of surface wave excitation can work even when the peak temperature in the melt pool is below the boiling point. Experiments showing the relatively large removal rate at 8 kHz compared to the unmodulated CW case support this conclusion. Simulations of melt pool oscillation and ejections agrees well with high speed video of liquid droplet ejections. We show using confocal microscopy and CT scan that the energetic removal produces high quality aspect ratio hole of 12:1 with a very small heat affected zone that is largely free of surface cracks. The modulation technique can be applied to a wide variety of materials. The results presented here can stimulate further development of new techniques for laser drilling technology.

## Experimental Setup

A 600 W, 1080 nm fiber laser (JK lasers, model JK600FL) was used for the drilling experiments. The beam propagates through focusing optics to yield approximately 50, 75 and 100 μm 1/e^2^ at the sample. The setup includes a set of sample scanning stages to create tests matrices at different laser parameters. The laser was modulated at up to 40 kHz with a sinusoidal modulation. The modulation amplitude was 300 W at the sample with offset of 300 W. The gate was asynchronous with the modulation. The gating time was set at 5 ms for the ablation pits and off for deep drilling of holes. With modulation the power varied from 20 W to 600 W at the sample. Behind the sample, a pickoff mirror directs 0.5% of the light to a photodiode while the remaining transmitted beam is sent to a beam dump. The photodiode measures the drill-through time, defined as the time a signal is first detected with respect to the start of the laser. An ultra-high-speed imaging microscope is used to probe the melt pool. The imaging optics consist of a Mitutoyo 10X, 0.28 NA objective mounted on a Navitar microscope body with optical resolution of ~5 µm. The microscope angle is ~60 degrees for Supplementary Movie 1 and 2, and ~90 degrees parallel to the surface for Supplementary Movie 3. Recording is captured with either the Photron SA-X2 at 100 kfps or a Shimadzu HPV-2 camera at 200 kfps.

Incandescent emission dominates the signal received by the camera at the typical melting point of the samples. This prevents observation of the motion of the liquid. To solve this problem, an auxiliary light source (Cavitar, model Cavilux HF) at 808 nm is used to illuminate the sample.

A notch filter with a band pass window of 2 nm around the illumination wavelength blocks out the dominating incandescent, allowing the liquid to be easily observed. The illumination laser is synchronized with camera exposure and light level adjusted for best viewing to further reduce incandescence. The entire system, including stage motion, laser positioning, triggering and camera or detector synchronization is automated using LabVIEW.

## Materials

Al6061 bare plate substrates were obtained from McMaster Carr and machined into 1″ diameter discs of 1 or 2 mm thickness. The discs were bead blasted on the side to prevent specular reflections.

## Supplementary information

Comparison of laser drilling at different modulation frequencies

Resonant amplification over three cycles

Macroscopic view of laser drilling viewed from side
